# Effective and Timely Evaluation of Pulmonary Congestion: Qualitative Comparison Between Lung Ultrasound and Thoracic Bioelectrical Impedance in Maintenance Hemodialysis Patients

**DOI:** 10.1097/MD.0000000000000473

**Published:** 2015-02-13

**Authors:** Carlo Donadio, Laura Bozzoli, Elisa Colombini, Giovanna Pisanu, Guido Ricchiuti, Eugenio Picano, Luna Gargani

**Affiliations:** From the Department of Clinical and Experimental Medicine, Division of Nephrology, School of Nephrology, University of Pisa, Pisa, Italy (CD, LB, EC, GP, GR); Institute of Clinical Physiology, National Research Council, Pisa, Italy (EP, LG).

## Abstract

The assessment of pulmonary congestion in maintenance hemodialysis (MHD) patients is challenging. Bioelectrical impedance analysis (BIA) can estimate body water compartments. Natriuretic peptides are markers of hemodynamic stress, neurohormonal activation and extracellular volume overload. Lung ultrasound (LUS) has been proposed for the non-invasive estimation of extravascular lung water through B-lines assessment. Up to now, no study evaluated the correlation between B-lines, segmental thoracic BIA, and natriuretic peptides in MHD patients. The aims of this study were: (1) To validate LUS as a tool for an effective and timely evaluation of pulmonary congestion in MHD patients, in comparison with segmental thoracic BIA, and with natriuretic peptides; (2) To compare a comprehensive whole chest ultrasound scanning with a simplified and timely scanning scheme limited to the lateral chest regions.

Thirty-one MHD adult patients were examined. LUS, total body and thoracic BIA, and natriuretic peptides were performed immediately before and after a mid-week dialysis session. The number of B-lines assessed by LUS was compared with total body and thoracic impedance data and with natriuretic peptides.

Pre-HD B-lines ranged 0–147 (mean 31) and decreased significantly post-HD (mean 16, *P* < 0.001). A significant correlation was found between the number of B-lines and extra-cellular water index (ECWI, *r* = 0.45, *P* < 0.001), with thoracic impedance (*r* = 0.30, *P* < 0.05), and with BNP (*r* = 0.57, *P* < 0.01). The dynamic changes in B-lines correlated better with thoracic impedance than with total body impedance, and correlated with extra-cellular but not with intra-cellular water index. The correlation between B-lines and ECWI was similar when LUS was limited to the lateral chest regions or performed on the whole chest. Multivariate analysis showed that only segmental thoracic impedance was an independent predictor of residual pulmonary congestion.

The dynamic changes in B-lines after hemodialysis are correlated to the changes in total body and extra-cellular water, and particularly to lung fluids removal. B-line assessment in MHD patients is highly feasible with a simplified and timely scanning scheme limited to the lateral chest regions. These premises make B-lines a promising biomarker for a bedside assessment of pulmonary congestion in MHD patients.

## INTRODUCTION

Dyspnea due to pulmonary congestion is frequent in maintenance hemodialysis (MHD) patients. The increase in extravascular lung water (EVLW) can be related to total body extracellular volume overload or to cardiac dysfunction. Both conditions are frequent in MHD patients. The evaluation of “ideal” body weight is a key point in dialysis treatment and can influence the outcome of patients.^1,2^ The clinical assessment of ideal body weight and EVLW may frequently be imprecise, allowing both subclinical conditions of hyper- and hypo-hydration. The ideal method for assessing ideal body weight and EVLW should be reliable, simple, non-invasive, inexpensive and feasible for the repeated evaluations. Different methods have been proposed, such as evaluating natriuretic peptide levels,^3,4^ dimension and collapsibility of the inferior vena cava,^5^ chest X-ray signs, and bioelectrical impedance analysis techniques.^6,7^ However each method has significant theoretical and practical limitations. More recently, lung ultrasound (LUS) has been validated for the semi-quantification of pulmonary congestion, through the assessment of B-lines, the sonographic sign of the pulmonary interstitial syndrome.^8^ Commonly, LUS is performed by scanning the antero-lateral—and possibly the posterior—regions of the chest. In patients with heart failure, an increased number of B-lines correlate with the degree of extravascular lung water,^9^ and a decreased number of B-lines mirrors the efficacy of treatment.^10^ Previous studies have shown that LUS can detect EVLW and its significant reduction after a dialytic session, both in HD and in peritoneal dialysis.^11–14^ Existing data suggest that LUS characteristics may be suitable for the assessment of ideal body weight in MHD patients, since this technique is simple, inexpensive, non-ionizing and easily available at the bedside.^15,16^ However, conflicting results have been reported on the correlation between B-lines number and the volumes of body water compartments, evaluated by total body BIA.^11,17^ Furthermore, up to now no study evaluated the correlation of B-lines with segmental thoracic BIA and with natriuretic peptides in MHD patients. For these reasons we planned the present study to validate LUS, as a safe tool for an effective and timely evaluation of pulmonary congestion in MHD patients, in comparison with segmental thoracic BIA. The dynamic changes during HD in B-lines were compared with those in total body and segmental thoracic BIA and in natriuretic peptides to assess the correlation of LUS findings with the volumes of total body and thoracic fluids and with serum levels of markers of hearth dysfunction. In the mean time, the comprehensive and time-consuming antero-lateral and posterior ultrasound scanning scheme to assess B-lines was compared with a simplified and timely scanning limited to the lateral chest regions.

## METHODS

### Study Population

Between April and September 2012, all 40 MHD patients treated during that period in the Dialysis Unit of the Nephrology Division of the University of Pisa were evaluated. Exclusion criteria were: age <18 years, massive pleural effusion, severe chronic obstructive pulmonary disease, pneumothorax, pulmonary fibrosis, very compromised general conditions and patients unable to give informed consent. Nine patients had 1 or more exclusion criteria. The remaining 31 MHD patients were enrolled in the study. Two patients were studied twice, 1 month after the change in their dialysis schedule (1 patient from 1 to 2 sessions/week, the other one from twice to 3 times/week). One patient did not complete the post-HD examination due to severe intra-dialytic hypotension. The study was approved by the Institutional Review Board at Pisa University Hospital (protocol no. 33954). All patients provided written informed consent.

The following clinical, laboratory and instrumental parameters were measured immediately before (pre-HD) and at the end (post-HD) of a mid-week dialysis session. Data were immediately recorded on individual case report forms and stored in a file excel. Check for eventual errors in the records was made in duplicate.

### Clinical Evaluation

Lung auscultation, arterial blood pressure, heart rate, body weight, height, body mass index, daily residual diuresis, NYHA class were recorded. Symptomatic intra-dialytic hypotension, any other disorder and administration of fluids or drugs during dialysis session were recorded.

### Laboratory Parameters

Serum urea, creatinine, albumin, C-reactive protein and blood count were measured with standard laboratory methods. B-type natriuretic peptide (BNP) (Triage BNP, Biosite, CA, USA) and N terminal pro-B-type natriuretic peptide (NT-proBNP) (pro BNP II, ECLIA, Cobas), were measured following standard procedures. Dialytic efficiency (spKt/V) was evaluated with single pool variable volume Kt/V formula from dialysis length, serum urea concentrations and body weight before and after dialysis.^18^

### Instrumental Parameters

#### Body Impedance Analysis (BIA)

Total body electrical impedance to an alternate current (0.2 mA) with 4 different frequencies (5, 50, 100, and 200 KHz) was measured using a multi-frequency analyzer (Quadscan 4000, Bodystat, UK). Impedance at the 4 different frequencies was measured.

#### Total Body BIA

Four electrodes were placed on the right hand and foot,^19,20^ or on the side contralateral to the vascular access, with patients in the supine position. Total body water (TBW) and extra-cellular water (ECW) volumes were derived from electrical measurements combined with body weight, height, age and gender on the basis of the manufacturers equations. TBW and ECW volumes were indexed to squared height of the patients, obtaining total body water index (TBWI) and extra-cellular water index (ECWI).

#### Thoracic BIA

Two electrodes were positioned on the left and right sides of the neck and 2 on each hemitorax along mid-axillary lines, at the level of the xiphoid process. Impedance values were recorded for each hemi thorax.

#### Lung Ultrasound

**FIGURE 1:**
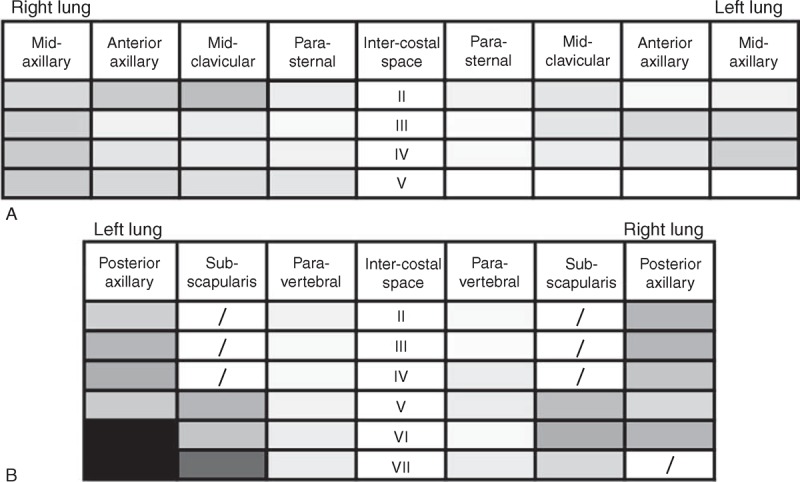
(A and B) Lung ultrasound scanning schemes with gray levels corresponding to mean number of B-lines pre-dialysis: antero-lateral (A) and posterior (B) scanning sites. Darker colors correspond to higher mean B-lines number.

Lung ultrasound was performed using a commercially available echographic system (MyLab 30, Esaote Biomedica, Genova, Italy) equipped with a 3.5–5 MHz convex transducer. The LUS examinations were performed with patients in the supine or near-supine position for antero-lateral scanning, and in the sitting position for dorsal scanning, as previously described.^21,22^ The anterolateral chest was scanned on a total of 28 scanning sites on the right and left hemithorax, from the second to the fourth (on the right side to the fifth) intercostal spaces along the parasternal, mid-clavicular, anterior axillary, and mid-axillary lines (Figure 1A). We scanned the posterior chest on a total of 29 scanning sites on the right and left hemithorax, along the paravertebral, scapular and posterior axillary lines; we started from the apex and proceeded downwards until the diaphragm was visible (Figure 1B). The total number of chest sites scanned was 3648 (57 scanning sites per patient pre-HD and post-HD). A video showing how to perform a whole LUS examination in detail can be freely accessed at the following link: http://www.youtube.com/watch?v=amsULLws8GI.

A B-line was defined as an echogenic, coherent, wedge-shaped signal with a narrow origin in the near field of the image.^8^ The number of B-lines was recorded in each intercostal space. The sum of B-lines created a score indicating the degree of EVLW. Zero was defined as a complete absence of B-lines. The full white screen in a single scanning site was considered as corresponding to 10 B-lines. A total number of B-lines >30 was assumed, according to previous data, as marker of clinically relevant pulmonary congestion.^23^

The intra- and inter-observer variability of B-line assessment had been previously established as 5.1% and 7.4%, respectively.^21^ The inter-observer agreement has been found to be very high also in MHD patients.^11,24^

Total body BIA, segmental thoracic BIA and lung ultrasound were performed immediately before and 30 min after the dialytic session. The complete procedure takes approximately 30 min at each time point. While performing each test, the authors were unaware of the results of the other tests.

#### Echocardiography

Thirty patients underwent a comprehensive transthoracic echocardiography examination at rest, once in a short interdialytic interval, with a commercially available ultrasound system (Toshiba Aplio XG) equipped with a 2.5–3.5 MHz phased-array sector scan probe, second harmonic technology, and coupled with tissue Doppler imaging (TDI). The inferior vena cava (IVC) diameter and collapsibility index were also measured during the echocardiographic examination in all patients, when feasible. All echocardiographic measurements were performed according to the recommendations of the European Association of Echocardiography/American Society of Echocardiography by a cardiologist unaware of the LUS and BIA results.^25–27^

### Statistical Analysis

Continuous variables are expressed as mean ± standard deviation. Categorical variables are presented as counts and percentages. Correlations between parameters were assessed with parametric Pearson or non-parametric Spearman correlation coefficient analysis, as appropriate. Differences between independent samples or between paired samples were tested using the non-parametric tests Mann–Whitney and Wilcoxon, respectively, and by Kruskal–Wallis test. The association of selected variables with the presence of B-lines was assessed by logistic regression analysis using univariate and stepwise multivariate procedures. A *P*-value <0.05 was considered statistically significant. Statistical analysis was performed using IBM SPSS Statistics (version 20.0, SPSS, Inc., Chicago, IL), MedCalc® Software Version 12.4.0.0 (Mariakerke, Belgium), and GraphPad Prism (version 6 GraphPad Software, Inc., San Diego, CA).

## RESULTS

Demographic and clinical characteristics of the 31 examined patients are reported in Table 1.

**TABLE 1:**
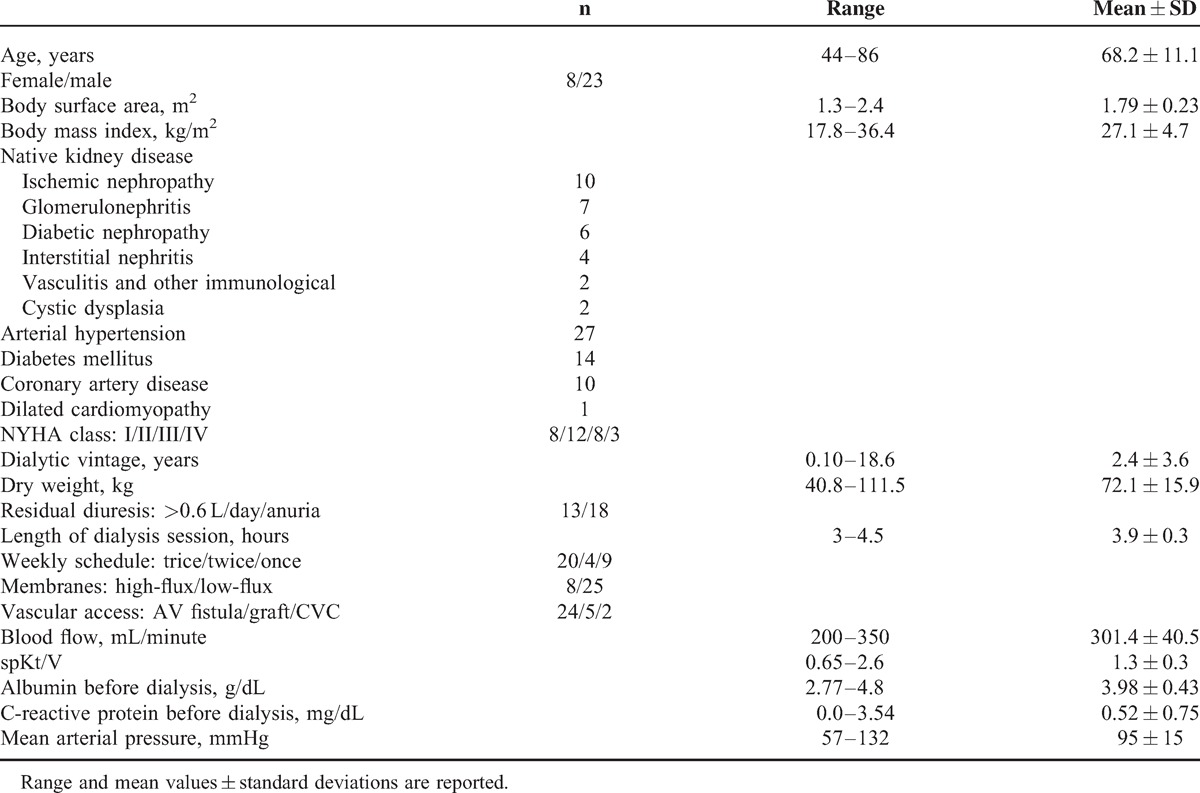
Demographic, Clinical, and Dialytic Characteristics of the 31 Patients Enrolled

### Dialysis Treatment

The effects of dialytic treatment on body weight, LUS, natriuretic peptide levels, and total body and thoracic BIA are reported in Table 2.

**TABLE 2:**
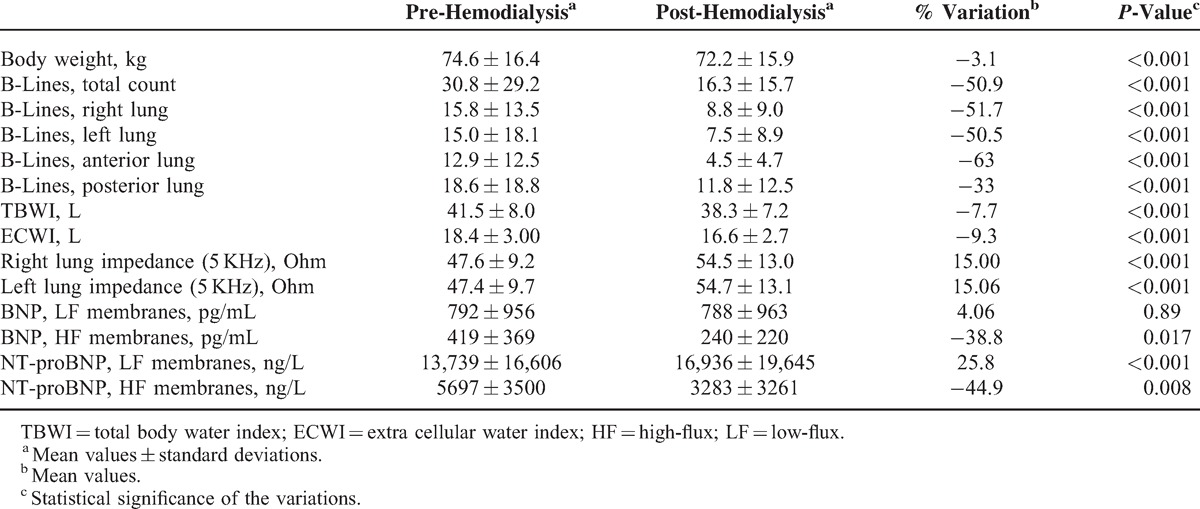
Effect of Dialysis Treatment on Sonographic, Biochemical and Instrumental Parameters

Body weight of patients decreased from 74.6 ± 16.4 to 72.2 ± 15.9 kg post-HD (−3.1%, *P* < 0.001). Mean arterial blood pressure did not significantly change (95 ± 15 before HD vs 98 ± 13 mmHg after HD, *P* = 0.13).

### Lung Ultrasound

**FIGURE 2:**
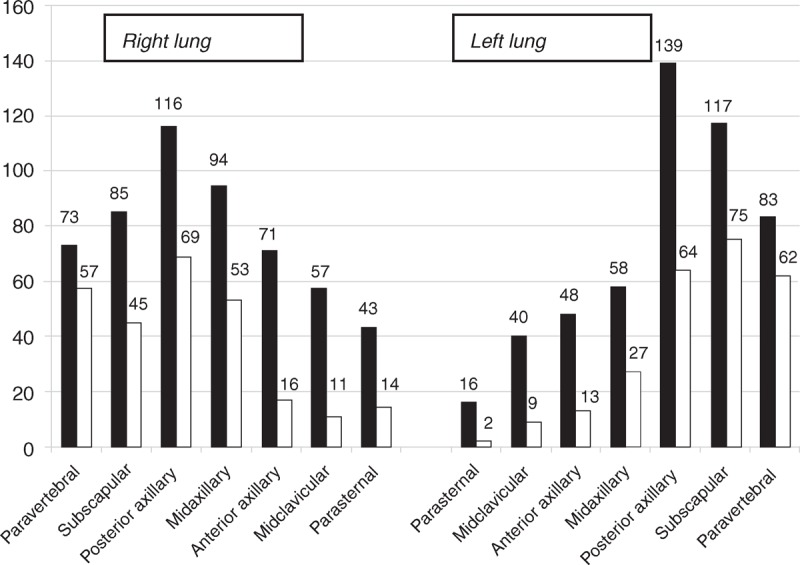
Lung ultrasound: frequency distribution of the B-lines along the different thoracic lines before (solid bars) and after (empty bars) the hemodialysis session.

**FIGURE 3:**
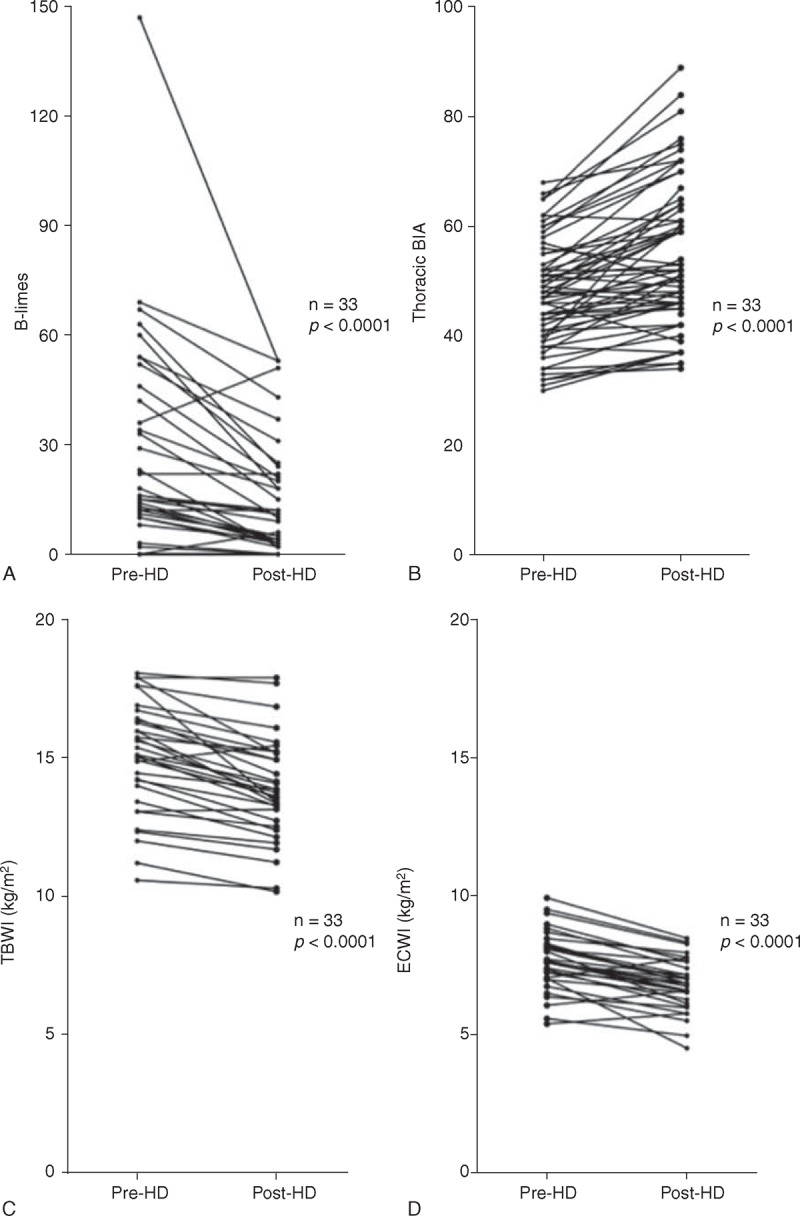
(a–d) Dynamic variation after hemodialysis in the number of B-lines, evaluated by lung ultrasound and for comparison, in thoracic impedance, in total body water index (TBWI), and in extra-cellular water index (ECWI). Mean values ± SD are represented.

Feasibility of LUS was 100%. Pre-HD B-lines were more numerous on posterior chest compared to anterior chest (18.6 ± 18.8 vs 12.9 ± 12.5, *P* < 0.05), and on the axillary lines compared to the parasternal lines (15.9 ± 14.8 vs 4.7 ± 5.5) (Figure 1A and B). The reduction in number of B-lines post-HD was significantly correlated to the reduction in body weight (*r* = 0.39, *P* < 0.05). Pre-HD B-lines ranged between 0 and 147 (mean value 31), and decreased significantly by 51% (*P* < 0.001) post-HD (Figures 2 and 3a). Pre-HD 6 patients had absence or trivial degree of B-lines (0–10 B-lines), 14 patients had mild degree (11–30 B-lines), 9 patients had moderate degree (31–60 B-lines), and 4 patients had severe degree (>60 B-lines). Post-HD 15 patients had absence or trivial degree of B-lines, 12 patients had mild degree, 6 patients had moderate degree, and no patient had severe degree. The number of post-HD B-lines was significantly correlated with the number of B-lines pre-HD (*r* = 0.82, *P* < 0.001). The total number of B-lines pre- and post-HD, and hence their reduction, was similar in the right and left lung (Table 2, pre-HD right 15.8 ± 13.5 vs left 15.0 ± 18.1, *P* = 0.25; post-HD right 8.8 ± 9.0 vs left 7.5 ± 8.9, *P* = 0.17). A greater reduction in B-lines post-HD was found in the antero-lateral lung segments compared to the posterior segments (−63% vs −33%, Table 2 and Figure 2). No significant correlation was found between number of B-lines and either presence of crackles at lung auscultation, or with NYHA functional class of patients.

### Echocardiographic Findings

A thorough echocardiogram was obtained in 30 patients, once in a short interdialytic interval. Left ventricular ejection fraction (LVEF) ranged between 33% and 73% (mean 60 ± 8%), and was normal (>55%) in 28/30 patients. Diastolic function was normal in 4/30 patients, mildly abnormal (impaired LV relaxation) in 16/30, moderately abnormal (pseudo normal pattern) in 9/30 and severely abnormal (restrictive pattern) in 1/30. All other echocardiographic data are listed in Table 3. Interestingly enough, B-lines post-HD correlated with *E*/*e*′ (*r* = 0.34, *P* < 0.05), LVEF (*r* = −0.59, *P* < 0.001), and pulmonary artery systolic pressure (PASP) (*r* = 0.53, *P* < 0.01), whereas B-lines pre-HD did not correlate with the same parameters. No correlation was found between the number of B-lines (both pre- and post-HD) and the other measured echocardiographic parameters and inferior vena cava (IVC) diameter.

**TABLE 3:**
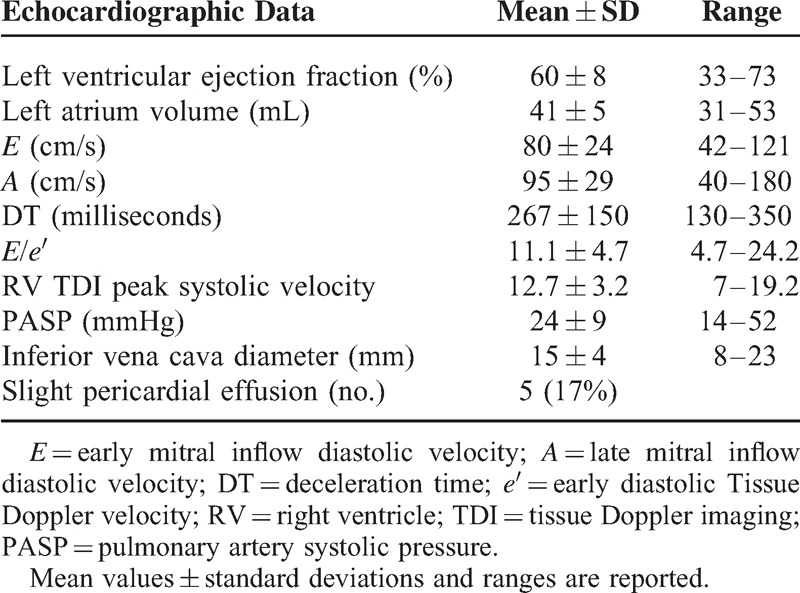
Echocardiographic Data Obtained Once in a Short Interdialytic Interval

### Natriuretic Peptides

Pre-HD natriuretic peptide values were markedly increased in nearly all patients. Pre-HD BNP and NT-proBNP were higher in patients treated with low-flux (LF) membranes than in those treated with high-flux (HF) membranes (Table 2). Indeed, only the treatment with HF membranes removed BNP and NT-proBNP from plasma, by approximately 40%. A significant correlation was found between the pre-HD plasma concentrations of natriuretic peptides and LVEF (*r* = 0.55, *P* < 0.01), as well as with *E*/*e*′ (BNP *r* = 0.57, *P* < 0.01; NT-proBNP *r* = 0.45, *P* < 0.01). A significant correlation was found between BNP values and B-lines post-HD (*r* = 0.57, *P* < 0.01), but not with B-lines pre-HD.

### Total Body BIA and Segmental Thoracic BIA

Thoracic impedance, measured at 5 kHz, was significantly correlated with total body impedance (left lung *r* = 0.4, *P* = 0.001; right lung *r* = 0.38, *P* = 0.002). The decrease in number of B-lines post-HD was accompanied by the decrease in TBWI and ECWI, and by the increase in thoracic impedance, indicative for a decrease in lung water content (Figure 3a–d). A significant correlation between the number of B-lines and the lung water content estimated by BIA was found on both sides (right lung impedance *r* = 0.30, *P* < 0.05; left lung impedance *r* = 0.29, *P* < 0.05). A significant correlation was found between the total number of B-lines and the TBWI (*r* = 0.34 *P* < 0.001), and a stronger correlation was found with ECWI (*r* = 0.45, *P* < 0.001). The topographic analysis of different chest regions revealed that although posterior scanning sites tend to show a higher number of B-lines, the correlation of B-lines with TBWI and ECWI is similar when the total number of B-lines is calculated anteriorly or posteriorly (Table 4). No significant correlation was found between number of B-lines and intra-cellular water index (ICWI). Lung impedance also correlated with natriuretic peptide values (*r* = 0.39, *P* < 0.001). There was a trend in the correlation between IVC diameters and lung impedance, although it was not statistically significant (*r* = −0.37, *P* = 0.06). TBWI and ECWI were not correlated to any echocardiographic parameter, to IVC diameters, nor to natriuretic peptide values. Univariate and multivariate analysis showed that only lung impedance is an independent predictor of the presence of more than 30 B-lines post-HD (Table 5).

**TABLE 4:**
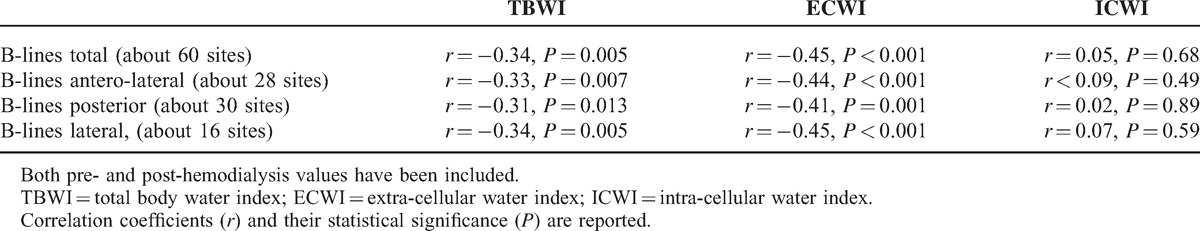
Correlation Between Body Water Compartments by Total Body BIA and Number of B-Lines at Lung Ultrasound Examinations

**TABLE 5:**
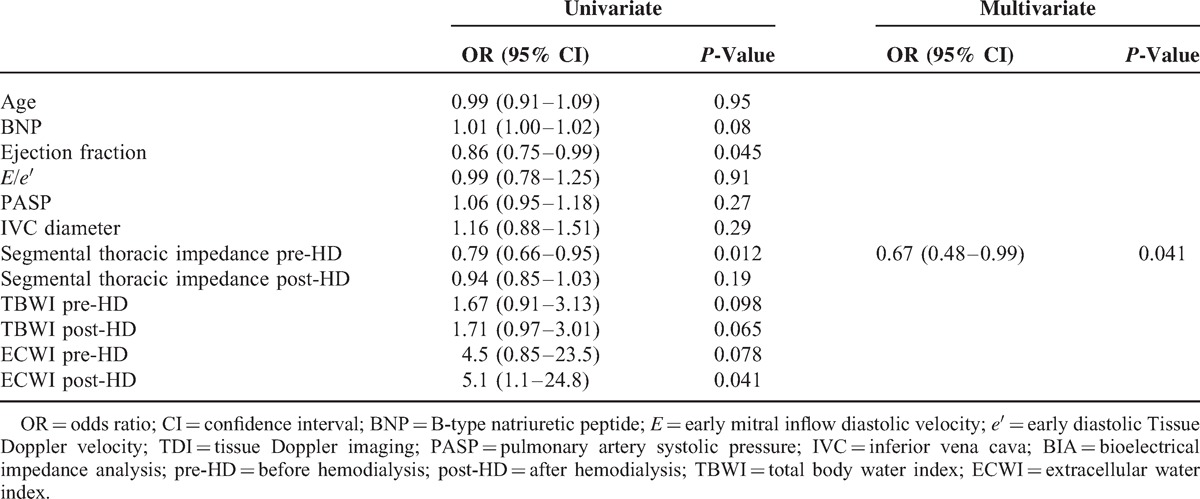
Univariate and Multivariate Analysis to Predict Post-HD B-Lines > 30

## DISCUSSION

The present study was aimed to validate LUS as a simple tool for an effective and timely bedside evaluation of pulmonary congestion in MHD patients, in comparison with total body and segmental thoracic BIA, and with natriuretic peptides. The major advantages of BIA are its simplicity of use and low cost, the possibility of bed-side examinations, and the high repeatability of measurements (7). A possible cause of errors is the use of prediction equations to estimate body compartments from electrical measurements of BIA. It is relevant to note that all the data of segmental thoracic BIA have been obtained exclusively on the basis of the electrical measurements.

The study group was quite small but homogeneous, and a comprehensive analysis of the parameters indicative for lung congestion was performed in all patients. In our clinically stable MHD patients we found a significant correlation between LUS B-lines, total body BIA and with segmental thoracic BIA. This is the first study to correlate ultrasound B-lines to segmental thoracic BIA in MHD patients. Previous data in cardiological and intensive care settings have shown that LUS is reliable for assessing EVLW compared to the thermodilution technique,^9^ gravimetric method,^28,29^ chest X-ray, ^21^ or chest computed tomography.^30^ More recently, LUS has been proposed in MHD patients as a new tool for assessing the achievement of ideal body weight.^11,13,24^ A correlation between the reduction of B-lines and decrease in body weight during hemodialysis was found, suggesting the usefulness of LUS in evaluating decreased ECW.^12^ In MHD patients, the number of B-lines pre-HD is correlated with increased interdialytic body weight; the reduction in B-lines correlates with decreased body weight during HD, and the residual B-lines post-HD are correlated with residual increase in body weight, independently of patient's symptoms.^13,31^ A correlation between the number of B-lines and pulmonary congestion was also found in peritoneal dialysis patients,^14^ where, similarly to MHD patients, B-lines and not NYHA class are independent predictors of physical functioning measured by the Kidney Disease Quality of Life Short Form.^32,33^

The number of B-lines and their dynamic changes with HD resulted correlated with ECW volume and with IVC diameters and collapsibility.^24^ Finally, 2 recent studies indicate that B-line score is a strong independent predictor of death and cardiac events in MHD patients, performing better than BIA itself.^17,31^

Our data are substantially consistent with Mallamaci et al^11^ and Siriopol et al^17^ about the correlation between LUS and EVLW. In our patients LUS correlated also with TBW and ECW estimated by BIA. However, when performing multivariate analysis, in our population only thoracic impedance and not total body BIA was an independent predictor of the presence of a significant number of residual post-HD B-lines. It is also to be acknowledged that the study populations are rather different, with normal systolic function in nearly all patients in our study, and a significant number of patients with systolic dysfunction in Mallamaci et al.

The whole thoracic ultrasound scanning indicates that the distribution of B-lines is not homogeneous in the different lung segments. A higher number of B-lines was indeed found in the lateral and posterior regions, which roughly correspond to the dependent zones, according to the position of the patient. The dynamic changes in B-lines post-HD were also not homogeneous, being more evident in the antero-lateral chest than in the posterior chest. However, the correlation with TBWI and ECWI found in the different lung segments appear fairly similar. These data could be translated into clinical practice, limiting the LUS examination to a shorter assessment of lateral chest regions focused along the axillary lines. It is true that further prospective studies with larger populations are needed and welcome to confirm the possibility to narrow the number of sites for LUS scans. However, although the number of patients was rather small, the measurements were performed in a group of patients who share similar characteristics and disease expression.

An increase in B-lines was also observed in patients without clinical findings, such as lung crackles, underlining, as previously reported, the discrepancy between presence of pulmonary congestion and clinical symptoms that tends to appear in more severe degrees.^34^

The high concentrations of natriuretic peptides, which are found in hemodialysis patients, may be due to increased secretion, reduced renal clearance and volume expansion.^35,36^ The marked increase in BNP and NT-proBNP values found in nearly all our patients seems mainly due to advanced impairment in renal function. As also indicated by other studies, NT-proBNP was more affected than BNP by the decline in renal function, probably due to its exclusive renal clearance.^3^ Dialytic treatment with LF or HF membranes affects differently the removal of natriuretic peptides both in pre-dialysis concentrations and even more in post-dialysis plasma values.^37–39^ In fact, only HF membranes can remove BNP and NT-proBNP from plasma. It is interesting to note that whereas natriuretic peptides correlate with echocardiographic parameters, such as *E*/*e*′ and PASP both pre- and post-HD, B-lines correlate with the same parameters only post-HD. We may speculate that this different behavior underlines the different pathophysiological meaning of the 2 entities: natriuretic peptides can be considered a biomarker of hemodynamic overload, whereas B-lines are more specifically a biomarker of EVLW, thus representing pulmonary not hemodynamic congestion, which persists post-HD only in a minority of patients.

Recent data, from an experimental model of heart failure, demonstrated that thoracic impedance values correlate negatively with the severity of heart failure and with left atrial pressure, suggesting lung congestion.^40^ We showed that both LUS and segmental thoracic BIA can estimate EVLW content and its variations. Our data confirm that EVLW increase, evaluated by LUS, is linked to the increase in extra-cellular body water. Moreover, EVLW increase is more closely linked to the increased lung water content, indicated by thoracic impedance, than total body water content. Although LUS seems very reliable to assess pulmonary congestion and its variation, it may be suboptimal, as a stand-alone tool, to more accurately determine the ideal dry weight in HD patients. LUS is indeed able to detect EVLW, but when the lung is not congested, LUS just shows a normal pattern, and cannot differentiate a dry from a “too dry” patient, or a euvolemic patient without lung congestion from a patient without lung congestion but with still significant systemic congestion. Of course, as for any patient's management, an integrated evaluation starting from the clinical picture and including different biomarkers is advised, rather than relying only on 1 parameter.

We should acknowledge some limitations of the study. (1) It is a single-center study with a relatively small number of enrolled patients. However, since we scanned almost 60 scanning sites per patient pre- and post-HD, the final total number of chest sites available for the statistical analysis was high (3648 assessments). (2) Being an ultrasound evaluation, LUS shares all limitations related to an operator-dependent technique. It is also true that since the examination is much simpler than other ultrasound applications (ie, echocardiography, abdominal ultrasound, etc.), the inter-operator variability is low.^11,16,21,24^ (3) B-lines are a non-specific sign of pulmonary interstitial syndrome; therefore they can be visible also in different conditions, such as pulmonary fibrosis, acute respiratory distress syndrome, interstitial pneumonia. In our population we excluded patients with these significant pulmonary conditions. In larger populations in the clinical arena, this lack of specificity may pose some issues, especially when the pre-existent pulmonary condition is not known. It is especially in patients with pulmonary fibrosis or other pulmonary interstitial syndromes that B-lines cannot be used to reliably evaluate pulmonary congestion.

## CONCLUSIONS

Our data show that (1) B-lines assessment in MHD patients is highly feasible. Furthermore, an ultrasound-focused examination of the lateral chest regions appears to be as accurate as the more comprehensive antero-lateral and whole chest assessments. (2) The dynamic changes in B-lines due to hemodialysis are correlated to the changes in total body and extra-cellular water and particularly to lung fluids content. These premises make B-lines a promising simple biomarker that could help in the management of pulmonary congestion of MHD patients.
